# Prevalence of HIV and Syphilis Infection among Men Who Have Sex with Men in China: A Meta-Analysis

**DOI:** 10.1155/2014/620431

**Published:** 2014-04-23

**Authors:** Yunhua Zhou, Dongliang Li, Dabing Lu, Yuhua Ruan, Xiao Qi, Ge Gao

**Affiliations:** ^1^Department of Epidemiology and Health Statistics, School of Public Health, Suzhou University, Suzhou, Jiangsu 215123, China; ^2^Chaoyang Center for Disease Control and Prevention, Beijing 100021, China; ^3^State Key Laboratory for Infectious Disease Prevention and Control, National Center for AIDS/STD Control and Prevention, Chinese Center for Disease Control and Prevention, Collaborative Innovation Center for Diagnosis and Treatment of Infectious Disease, Beijing 100026, China

## Abstract

*Objectives*. To figure out the most current prevalence of HIV and syphilis in MSM in China. *Methods.* A meta-analysis was conducted on the studies searched through PubMed, CNKI, and Wanfang published between 1 January 2009 and 11 April 2013. *Results.* Eighty-four eligible studies, either in Chinese or in English, were included in this review. The pooled prevalence of HIV and syphilis infection in MSM in China was 6.5% and 11.2%, respectively. The subgroup analyses indicated that the prevalence of HIV infection was higher in the economically less developed cities than that in the developed cities (7.5% versus 6.1%, *P* < 0.05). In contrast, the prevalence of syphilis infection was lower in less developed cities than in developed cities (8.6% versus 15.1%). Studies with a sample size smaller than 500 had a lower prevalence of HIV and syphilis infection than those with a sample size greater than 500 (5.9% versus 7.2% for HIV; 11.0% versus 11.5% for syphilis, respectively). *Conclusions.* HIV and syphilis infection are prevalent in MSM in China. The different prevalence of HIV and syphilis infection between developing and developed cities underscores the need to target prevention strategies based on economic conditions.

## 1. Introduction

The human immunodeficiency virus (HIV) and syphilis infection epidemics present an intractable problem to the world. This is also true in China, where there is a marked increase of people living with HIV/AIDS, from 740,000 in 2009 to 780,000 in 2011. Approximately 17.4% of new HIV cases are attributed to homosexual transmission [1]. It was said that the epidemic of HIV infection among men who have sex with men (MSM) has been the gravest of the six newborn HIV/AIDS challenges in China [2]. From 2009 to 2013, the prevalence of HIV among MSM ranged from 1.7% to 21.0% in China [3, 4]. A recent meta-analysis reported that almost 5.3% (95% CI: 4.8%–5.8%) of MSM in China were living with HIV, which was more than 90 times higher than the general public [5]. The prevalence of syphilis in MSM increased from 6.9% to 11.8% between 2003 and 2009 [6, 7].

The fast growth of the HIV epidemic has been observed in some reports [5, 8]. Although there have been several meta-analyses on prevalence of HIV and syphilis in MSM [5, 8, 9], the most recent meta-analysis only included studies until 2008. Thus, it is important for us to have a better and more thorough understanding of the current prevalence of HIV and syphilis. Therefore, we performed a meta-analysis of all available literature published from 2009 to 2013.

Given the poor awareness of AIDS-related knowledge in economically developing cities, which may lead to higher prevalence of marriage and bisexuality and lower condom use than economically developed cities, we also carried out a subgroup analysis to explore the differences in HIV and syphilis prevalence between developing and developed cities in China. Results from the analyses may guide future policies for more rational allocation of prevention and treatment resources.

## 2. Methods

### 2.1. Search Strategy

A systematic review of published articles was conducted by searching the following database: PubMed, Chinese National Knowledge Infrastructure (CNKI), and Wanfang from January 1, 2009, to April 11, 2013. Search terms included HIV, AIDS, syphilis, STD, sexually transmitted disease, prevalence, MSM, homosexual, gay, sex between men, and China. Reference lists were also referred to for additional articles. This review was conducted according to the Preferred Reporting Items for Systematic Review and Meta-Analyses (PRISMA) statement issued in 2009.

### 2.2. Study Selection

Studies were included if they met the following criteria: (1) published in Chinese or English language; (2) cross-sectional or cohort studies that reported either HIV or syphilis prevalence or both; (3) HIV and syphilis infection was diagnosed from at least two laboratory serologic testing methods; (4) articles that presented study design information, including study period, study site, and sampling method. Studies were excluded if (1) the study participants were MB (Money Boys) or young MSM (younger than 18 years old); (2) the sample size was smaller than 150. Studies with no specific study cities were also excluded from subgroup analyses. If articles with the same data were published in both Chinese and English, the English source was included.

### 2.3. Data Extraction

The first author extracted the following data from the included studies, which was later verified by the second author: the first author and published year, study period, study site, sampling methods, average age of MSM participants, sample size, and prevalence of HIV infection or syphilis infection or both. The study sites were divided into developing and developed cities (as assessed on the basis of based on per capita gross domestic product (GDP) for each city). Developing cities were defined as those with a per capita GDP lower than 15,000 RMB and developed cities were defined as cities with per capita GDP higher than 15,000 RMB. GDP of each city per person-year was obtained from http://tieba.baidu.com/p/2312163828. Disagreements were resolved by discussion. If no consensus could be reached, a third author would be referred to.

### 2.4. Validity Assessment

Validity assessments were accomplished by two workers. Studies were considered to be of high validity if they met the following criteria: (1) reported both HIV and syphilis infection; (2) used two or more recruitment methods; (3) sample size larger than 500; (4) published in English.

### 2.5. Statistical Analysis

Meta-analyses of the prevalence of HIV and syphilis infection among MSM were carried out by Meta-Analyst software (Beta 3.13). *Q*-test (significance level *P* < 0.10) and *I* ^2^ (significance level *I* ^2^ > 0.25) statistics were calculated to measure homogeneity of studies. Studies that had either *Q*  〈0.10  *or* *I* ^2^〉 0.25 were defined as heterogeneous. Potential publication bias was assessed by Begg's test. Pooled prevalence of HIV and syphilis infection, 95% confidence intervals, and the relative weight for each study were calculated by using random effects models. Due to the heterogeneity which is commonly observed in meta-analysis concerning sensitive groups such as MSM, we conducted a meta-regression with 10,000 permutations in a Monte Carlo simulation to explore the sources of between-study heterogeneity with the following covariates: economy status, sampling methods, sample size, and published year. Subgroup analyses were conducted to obtain the prevalences of HIV and syphilis from different stratifications.

## 3. Results

### 3.1. Study Selection for Meta-Analyses

**Figure 1:**
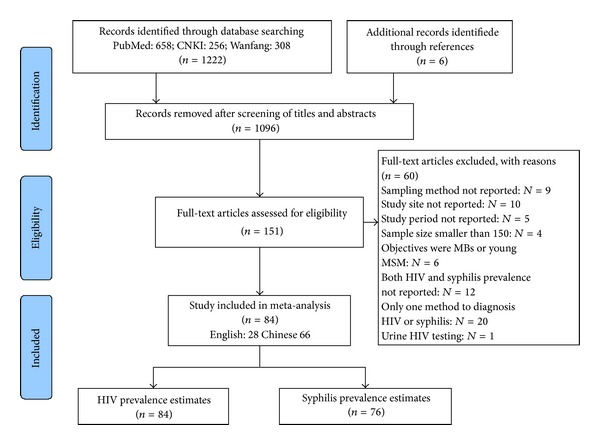
Flow chart of the meta-analysis studies selection.

1222 articles were retrieved after the initial search from databases and another 6 articles were included from reference lists. A total of 1096 articles were removed after screening of titles and abstracts because they were not topically relevant (*N* = 1077), were reviews (*N* = 7), and used the same data (*N* = 12). 151 articles were eligible for full-text screening and 67 were further excluded because they did not report the sampling methods or study site (*N* = 19), did not mention the study period (*N* = 5), had a sample size smaller than 150 (*N* = 4), included MB or young MSM (*N* = 6), presented neither HIV nor syphilis prevalence (*N* = 12), just used one method to diagnosis syphilis (*N* = 20), and tested HIV infection by urine (*N* = 1). The detailed process of data selection is illustrated by Figure 1.

### 3.2. Study Characteristic

Eighty-four studies with estimates of HIV prevalence (English 18, Chinese 66) and seventy-six studies with estimates of syphilis prevalence (English 17, Chinese 59) were finally identified in this review, which covers 59 cities from 29 provinces and municipalities in China (no studies from Hainan, Tibet, Hong Kong, Macao, and Taiwan). The prevalence of HIV and syphilis ranged from 1.7% to 21.0% and 0.9% to 31.2%, respectively [3, 4, 10–89]. In the meta-analysis of pooled prevalence of HIV, the sample size ranged from 150 to 6101 (total 71,845), and in the estimating of syphilis prevalence, the sample size oscillated between 157 and 6101 (total 65,162). About half of them were located in developing cities (50.7% and 51.4% in analyses of HIV and syphilis prevalence, resp.,). The following sampling methods were used to recruit participants: snow ball (*N* = 27), respondent driven sampling (RDS) (*N* = 15), multiple sampling methods (*n* = 23), and others (*N* = 19). The basic information of the 84 included studies is represented in Table 1.

### 3.3. Pooled Prevalence of HIV and Syphilis

**Figure 2:**
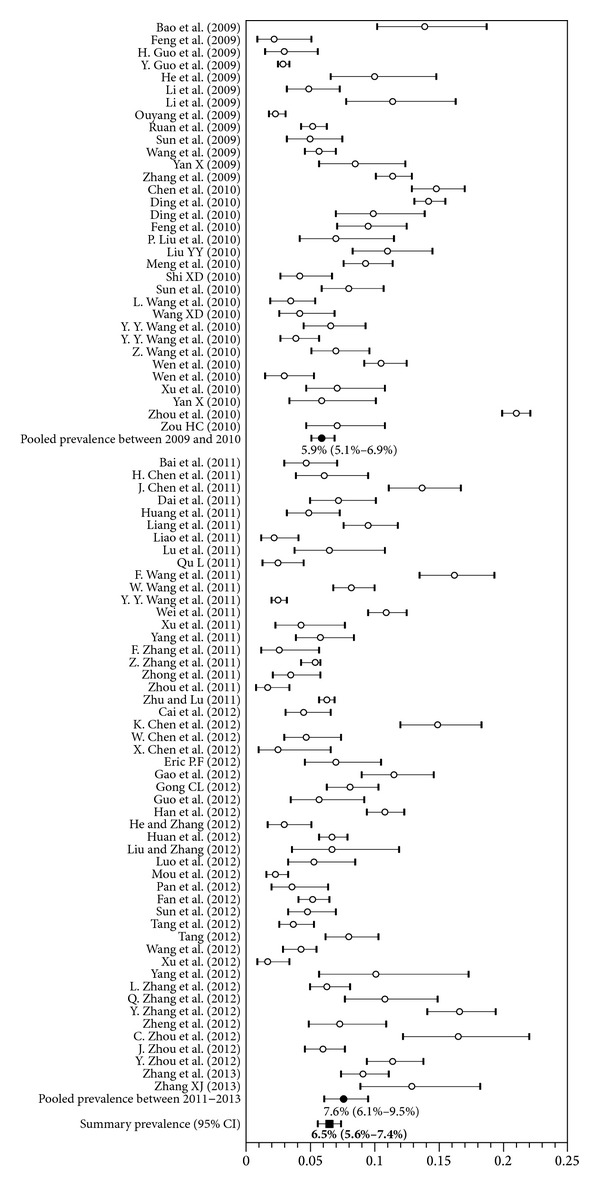
Forest plot showing the meta-analyses of pooled prevalence of HIV among men who have sex with men in China.

**Figure 3:**
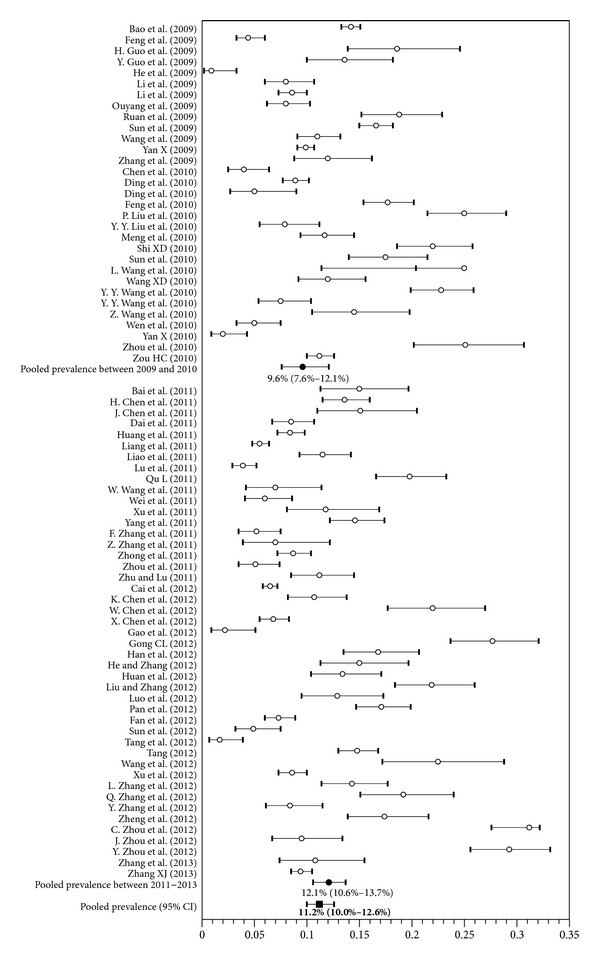
Forest plot showing the meta-analyses of the pooled prevalence of syphilis among men who have sex with men in China.

According to our meta-analyses, the estimates of HIV prevalence in MSM in China ranged from 1.7% to 21.0%. The pooled prevalence of HIV infection was 6.5% (95% CI 5.6% to 7.4%), as seen in Figure 2, with substantial heterogeneity (*P* < 0.001, *I* ^2^ = 0.490). Estimates of syphilis prevalence ranged from 0.9% to 31.2% and the overall prevalence was 11.2% (95% CI 10.0% to 32.6%), as shown in Figure 3, with substantial heterogeneity observed (*P* < 0.001, *I* ^2^ = 0.489).

### 3.4. Meta-Regression and Subgroup Analyses

Results of meta-regression indicated that economic status and sample size contributed to the heterogeneity between studies (Table 2).

Four and two studies were further excluded because of the absence of specific study cities in the subgroup analyses of HIV and syphilis prevalence by different economic status, respectively. The prevalence of HIV infection was slightly higher in developing cities (7.5%) than in developed cities (6.1%), whereas the prevalence of syphilis was significantly lower in developing cities (8.6%) than in developed cities (15.1%). Studies with a sample size smaller than 500 had a lower prevalence of HIV and syphilis infection than those with a sample size greater than 500 (5.9% versus 7.2%, 11.0% versus 11.5%, resp.) (Table 2).

Although the published year and sampling methods did not contribute to between-study variance, we also conducted subgroup analyses based on them. Although we only divided the published year into two groups, an uptrend of HIV infection and a decrease in syphilis infection were still observed (Table 2).

## 4. Discussion

This is an updated meta-analysis that presents the most current prevalence of HIV and syphilis infection in MSM in China. In order to obtain the prevalence from developing and developed cities, we also carried out a subgroup analysis by different economic status according to meta-regression. To our knowledge, this is the first study to compare the prevalence of HIV and syphilis infection between developing and developed cities in China. A national study survey of 61 cities by Wu reported the prevalence of HIV in MSM from the southwest, east, south, and northeast of China but did not account for the different economic conditions of each city [6].

Overall, eighty-four articles published between January 1, 2009, and April 11, 2013 were included in this review. It was observed that the prevalence of HIV and syphilis infection among MSM in China was 6.5% (95% CI 5.6% to 7.4%) and 11.2% (95% CI 10.0% to 12.6%), respectively, which was lower compared to other countries and cities. For instance, the prevalence of HIV among MSM was 14.2% in Brazil, 10.6% in Kenya, 21.5% in Senegal, 9.0% in Indonesia, 14.7% in India, and 24.6% in Thailand [120–123].

In spite of the relatively lower prevalence, there was evidence for the uptrend of HIV and syphilis infection among MSM in China. A meta-analysis of Chow reported that HIV infection among MSM has increased over the past years, from 1.4% (95% CI 0.8%–2.4%) during 2001–2003, to 2.3% (95% CI 2.0%–2.6%) during 2004–2006, and to 5.3% (95% CI 4.8%–5.8%) during 2007–2009 [8]. Results from our study indicate that the prevalence of HIV among MSM is still increasing. The expanding epidemic may be associated with the special role of MSM in China. Homosexuality is still not widely accepted by the general population. Therefore, marriage between MSM and women remains common, nn which case MSM might act as a bridge for HIV transmission to other MSM and the general population. It was reported that the prevalence of bisexual behavior among MSM in China was as high as 31.2% [124].

The subgroup analyses showed that the prevalence of HIV infection was 7.5% from developing cities and 6.1% from developed cities, whereas the prevalence of syphilis was 8.6% from developing cities and 15.1% from developed cities. The exact reasons for this finding are unclear, but one possible reason is that syphilis is a curable disease and several large scale public health programs were conducted in several developing cities in the past few years [74].

**Figure 4:**
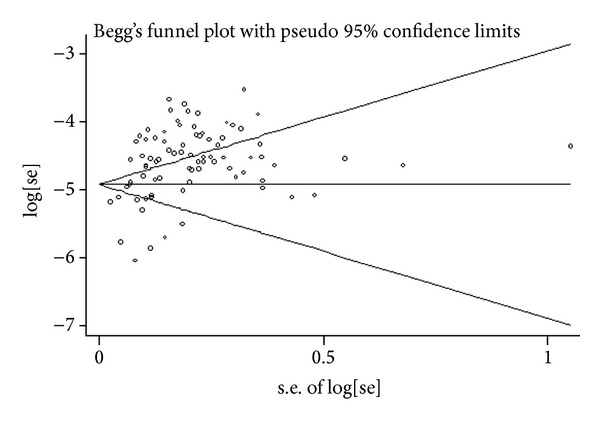
Begg's funnel plot showing the potential publication bias.

Several limitations of our analyses must be kept in mind. First, substantial heterogeneity was observed in our analysis. We found that study site, sample size, and sampling methods contributed to the heterogeneities. These factors may also have contributed to the increasing trend of HIV and syphilis infection in MSM in China. A meta-analysis of Chow reported that articles published in English were more likely to present high prevalence than Chinese articles, which may have resulted in between-study variance. The presence of publication bias was also a considerable limitation in our analysis (Figure 4). With the strict inclusion criteria, we excluded studies with a sample size smaller than 150 and those with only one testing method to diagnose syphilis. There are also a large number of unpublished articles (e.g., government documents) and studies that reported relatively low prevalence. Third, there were few studies from rural areas, which may have led to an overestimation of HIV and syphilis prevalence. More studies of rural MSM studies are needed in the future. Fourth, several studies that did not specify the city location were excluded from the subgroup analysis, which may have increased the possibility of publication bias. Fifth, the economic classification of the cities was only based on the per capita GDP; results may only partially reflect the true economic status of the city.

Despite the limitations listed above, our meta-analyses employed a strict inclusion criteria and valid search strategy in order to provide an objective, authentic, and current estimate of HIV and syphilis prevalence among MSM in China, with a large aggregate sample size of 75036. We also conducted a subgroup analysis to present the different prevalence rates of HIV and syphilis infection in developing and developed cities.

In conclusion, HIV and syphilis are prevalent among MSM in China; HIV prevalence is higher in developing cities than in developed cities, while the situation of syphilis is just the opposite. These results urgently indicate the need for strategies aimed at prevention, surveillance, and treatment. Moreover, corresponding policies should be drawn up by the local government on the basis of local economic status.

## Figures and Tables

**Table 1:** Meta-regression and subgroup analyses of HIV and syphilis prevalence among men who have sex with men in China.

Study characteristic	Stratified meta-analysis			
	HIV		Syphilis	
	Prevalence (95% CI)	Meta-regression (*β*, *P* value)	Prevalence (95% CI)	Meta-regression (*β*, *P* value)
Sample size				
<500	5.9% (5.0–7.0%)	1.017	11.0% (9.3–12.9%)	1.007
>500	7.2% (5.8–8.9%)	*P* = 0.056	11.5% (9.8–13.5%)	*P* = 0.670
Sampling methods				
RDS	7.4% (5.7–9.7%)	0.996	9.1% (7.1–11.6%)	1.009
Snow ball	7.2% (5.4–9.4%)	*P* = 0.356	13.3% (10.8–16.2%)	*P* = 0.198
Multiple Methods	5.9% (4.5–7.7%)		10.5% (8.2–13.3%)	
Others	6.2% (5.6–7.4%)		12.2% (10.0–14.9%)	
Economy status				
<15,000$	7.5% (6.5–8.6%)	0.971	8.8% (7.7–10.0%)	1.074
>15,000$	6.1% (4.7–8.0%)	*P* = 0.002	15.5% (13.0–18.3%)	*P* = 0.000
Published year				
2009-2010	5.9% (5.1–6.9%)	1.017	12.1% (10.6–13.7%)	−0.980
2011–2013	7.6% (6.1–9.5%)	*P* = 0.063	9.6% (7.6–12.1%)	*P* = 0.217

**Table 2:** Basic information of the 84 included studies.

First author, published year	Sample size	Sampling methods	Location	Economy status	Prevalence (%)	
					HIV	Syphilis
Liang, 2011 [35]	511	Multiple methods	Foshan	Developed	7.0	8.0
Sun, 2009 [50]	401	Multiple methods	Dalian	Developed	4.2	17.5
Yang, 2012 [72]	264	VCT	Fuyang	Developing	5.7	
Chen, 2012 [15]	400	Snow ball	Guilin	Developing	3.5	4.0
Wen, 2010 [64]	452	Snow ball	Guangzhou	Developed	4.9	6.0
Huang, 2011 [32]	395	Snow ball	Hefei	Developing	3.0	13.4
Wang, 2009 [54]	1348	Multiple Methods	Heilongjiang	Developing	2.3	14.8
Wang, 2010 [61]	200	Snow ball	Lanzhou	Developing	7.0	12.5
Wang, 2012 [58]	400	RDS	Mianyang	Developing	11.0	23.5
Lu, 2011 [40]	354	RDS	Nanchang	Developing	4.2	7.9
Chen, 2010 [3]	469	Snow ball	Nanning	Developing	1.7	10.7
Bao, 2009 [11]	6101	RDS	14 cities	Classified^a^	6.3	14.0
Chen, 2012 [16]	300	Snow ball	Yangzhou	Developed	7.3	22.0
Li, 2009 [34]	1773	Multiple methods	Chongqing	Developing	10.6	8.4
Guo, 2009 [27]	204	Venues-based	Tianjin	Developed	5.9	18.7
Liu, 2012 [90]	507	Snow ball	Changsha	Developed	4.3	25.1
Qu, 2011 [91]	805	Snow ball	2 cities	Developed	3.7	14.1
Chen, 2011 [12]	831	VCT	Kunming	Developing	11.4	13.6
Zhou, 2011 [83]	1166	Snow ball	Chongqing	Developing	14.8	8.7
Han, 2012 [92]	272	Venues-based	Taiyuan	Developing	8.5	13.6
Tang, 2011 [93]	408	Venues-based	Guilin	Developing	2.5	4.9
Xu, 2011 [69]	436	Venues-based	Liaoning	Dropped out^b^	3.0	5.0
Bai et al., 2011 [94]	280	Snow ball	Suzhou	Developed	7.1	15.0
Zhou, 2011 [95]	491	RDS	Chongqing	Developing	14.9	5.1
Fan, 2012 [47]	500	RDS	Beijing	Developed	8.0	22.0
Liao et al., 2011 [96]	2996	Multiple methods	Shandong	Developed	2.5	5.5
Feng et al., 2009 [97]	2044	Venues-based	Chongqing	Developing	11.5	8.9
He, 2009 [29]	423	RDS	Guangzhou	Developed	1.3	14.8
Eric, 2011 [98]	288	Snow ball	Yuxi	Developing	10.8	
Zheng, 2012 [81]	157	Multiple methods	Beijing	Developed	2.5	7.0
Wei, 2011 [63]	220	Snow ball	Taizhou	Developing	13.4	14.5
Zhong et al., 2011 [99]	379	RDS	Guangzhou	Developed	5.2	17.5
Zhang, 2012 [74]	503	RDS	Chongqing	Developing	15.7	6.6
Xiao, 2009 [66]	1692	Multiple methods	Chongqing	Developing	10.8	8.6
Xiao, 2010 [67]	4983	Multiple methods	20 cities	Dropped out^b^	2.9	9.8
Zhang et al., 2011 [100]	302	Snow ball	Beijing	Developed	9.9	19.2
Zhang et al., 2012 [101]	3314	Snow ball	Chongqing	Developing	14.2	9.4
Zhang, 2013 [75]	463	Snow ball	Harbin	Developing	9.5	14.3
Guo et al., 2009 [102]	433	Venues-based	2 cities	Developed	5.8	27.7
Ruan et al., 2009 [103]	541	Multiple methods	Beijing	Developed	4.8	19.8
Zou et al., 2010 [104]	429	Internet	2 cities	Classified^a^	4.8	11.4
Wang, 2011 [105]	2020	Surveillance	4 cities	Developing	2.3	
Gao, 2012 [106]	962	Multiple methods	Beijing	Developed	6.3	17.7
Zhou et al., 2010 [107]	550	Multiple methods	Beijing	Developed	4.5	
Zhou, 2012 [108]	294	VCT	Shanghai	Developed	6.1	9.5
Ouyang, 2009 [44]	617	RDS	Chongqing	Developing	16.8	10.9
Meng, 2010 [42]	287	Multiple methods	Changde	Developing	3.5	12.9
Tang, 2012 [52]	303	Multiple methods	Dazhou	Developing	3.6	1.7
Sun, 2012 [49]	2006	Multiple methods	Dalian	Developed	5.2	16.6
Dai, 2011 [109]	1315	VCT	Guangzhou	Developed	5.4	6.8
Mou, 2012 [110]	1526	Venues-based	Guangzhou	Developed	5.7	
Wen, 2010 [64]	452	Snow ball	Guangzhou	Developed	4.9	6.0
Wang, 2011 [111]	300	RDS	Kunshan	Developed	5.3	25.0
Sun, 2010 [112]	1350	RDS	2 cities	Developing	5.2	7.3
Zhu, 2011 [88]	259	Surveillance	Wuxi	Developed	13.9	25.1
Zhang, 2009 [76]	231	RDS	Urumqi	Developing	2.2	10.8
Chen, 2011 [113]	889	Snow ball	Lanzhou	Developing	6.0	13.6
Hong et al., 2009 [114]	593	Venues-based	Changzhou	Developed	13.7	31.2
Liu, 2012 [37]	661	Snow ball	Nanjing	Developed	3.9	11.5
He, 2012 [115]	235	Multiple methods	Geermu	Developing	4.3	0.9
Gong, 2010 [24]	252	Snow ball	Quanzhou	Developing	2.6	2.2
Zhang, 2012 [77]	300	RDS	Shenzhen	Developed	7.0	12.0
Pan, 2012 [45]	675	Multiple methods	Shenzhen	Developed	8.0	41.2
Shi, 2010 [116]	906	Venues-based	Shenzhen	Developed	4.2	14.5
Cai, 2012 [117]	5232	VCT	Shenzhen	Developed	6.5	21.0
Wang, 2010 [55]	900	RDS	Shenyang	Developed	9.3	11.0
Wang, 2011 [118]	450	Venues-based	Shijiazhuan	Developing	2.2	7.6
Wang, 2010 [61]	150	Snow ball	Shijiazhuan	Developing	6.7	11.3
Bai et al., 2011 [119]	280	Multiple methods	Suzhou	Developed	7.3	22.0
Zhang et al., 2011 [73]	717	Multiple methods	Suzhou	Developed	8.1	14.6
Xu et al., 2012 [70]	211	Multiple methods	Taizhou	Developing	10.0	11.8
Wang et al., 2011 [57]	201	Snow ball	Urumqi	Developing	6.5	7.0
Liu et al., 2010 [38]	456	RDS	Wuhan	Developed	6.6	21.9
Zhang et al., 2011 [80]	404	Snow ball	Xining	Developing	7.2	8.4
Wang et al., 2010 [62]	750	Multiple methods	Yangzhou	Developed	9.3	22.8
Chen et al., 2012 [14]	218	Snow ball	Chongqing	Developing	16.5	15.1
Yang et al., 2011 [71]	305	Multiple methods	Dali	Developing	3.0	2.0
Luo et al., 2012 [41]	1237	Snow ball	13 cities	Developing	8.2	3.9
Xu, 2010 [68]	1864	Multiple methods	4 cities	Classified^a^	6.7	
Ding et al., 2010 [19]	743	Snow ball	Chongqing	Developing	16.6	8.5
Ding et al., 2010 [20]	202	Snow ball	Chongqing	Developing	12.9	5.0
Li, 2009 [34]	1691	Multiple methods	Chongqing	Developing	10.9	8.6
Feng, 2010 [22]	946	Snow ball	Chongqing	Developing	15.8	
Guo et al., 2012 [25]	109	Surveillance	Zunyi	Developing	10.1	1.0

^a^Study sites were classified into different economy status when subgroup analysis was conducted; ^b^Study was dropped out then subgroup analysis was conducted.
